# Involving people with a lived experience when developing a proposal for Health Technology Assessment research of nonsurgical treatments for pelvic organ prolapse: Process and reflections

**DOI:** 10.1111/hex.13727

**Published:** 2023-02-13

**Authors:** Eugenie Evelynne Johnson, Joanne Lally, Allison Farnworth, Fiona Pearson

**Affiliations:** ^1^ Population Health Sciences Institute Newcastle University Newcastle upon Tyne UK; ^2^ Research Design Service North East and North Cumbria Newcastle upon Tyne UK; ^3^ NIHR Innovation Observatory, Faculty of Medical Sciences Newcastle University Newcastle upon Tyne UK

**Keywords:** HTA, patient and public involvement, pelvic organ prolapse, PPI

## Abstract

**Introduction:**

Patient and public involvement (PPI) is an expectation when conducting research, including Health Technology Assessment (HTA), but practical guidance for those wishing to embed PPI into the grant application process is not always easily accessible. We wanted to ensure that PPI was central when preparing a proposal for an investigator‐led evidence synthesis HTA investigating nonsurgical interventions for pelvic organ prolapse (POP) in women. Here, we describe our methods.

**Methods:**

We recruited two patient co‐applicants separately through an open process to help ensure that patient voice was present within our proposal's management and direction. We invited co‐applicants to attend research team meetings and comment on the full proposal. We designed, recruited to and facilitated a scoping workshop, as well as undertook its subsequent evaluation. The insight shared within the workshop for patients with a lived experience of POP, including our patient co‐applicants, helped us develop the scope and rationale behind our HTA proposal. We particularly considered the interventions to include within the evidence synthesis. We also considered the outcome measures for both the evidence synthesis and economic evaluation. We elicited ideas about where and how results could be disseminated. Feedback suggested the workshop was as valuable for the attendees as it was for the researchers, making them feel valued and listened to. The time spent by researchers working on the activity was substantial and not directly funded but a necessary and valuable activity in developing our potential HTA. Our work was informed using the UK Standards for Public Involvement and the Authors and Consumers Together Impacting on eVidencE (ACTIVE) framework.

**Conclusions:**

PPI can be enormously valuable in both developing and strengthening research proposals. However, further guidance is needed to help researchers recognise the level and type of involvement to use at this early stage, particularly given the large time investment needed to embed meaningful PPI.

**Patient and Public Contribution:**

Women with a lived experience of POP were involved at every stage of the grant application process; their involvement is documented in full throughout this work.

## 1 INTRODUCTION

Health Technology Assessment (HTA) is a multidisciplinary process that aims to determine the value of a health technology to inform decision‐making.^1^ In the context of HTA, health technologies mean any method to promote health and include procedures, drugs and devices, as well as diagnostic tests, settings of care and screening programmes.^2^ In the United Kingdom, the National Institute for Health Research (NIHR) state that HTA can include assessments made through systematic review, economic models, meta‐analyses and more.^2^ In the United Kingdom, the NIHR have a specific funding stream for HTA. Teams of researchers develop proposals for HTA, which are either commissioned specifically by the NIHR or are focused on topics identified as important by the researchers (researcher‐led topics). These proposals start as an initial, brief outline of the potential project and methods (Stage 1) and, if successful, are developed into more detailed applications (Stage 2) before being reviewed. Public and patient involvement (PPI) in proposal development is expected of researchers applying for health care funding, including for HTA, within the United Kingdom.

The UK Standards for Public Involvement measure and encourage greater PPI within research.^3^, ^4^ The first of these standards is ‘Inclusivity’, including the question: ‘Are people affected by and interested in the research involved from the earliest stages?’ Within the context of UK HTA proposals, involvement should begin before the Stage 1 application, so it can impact the research agenda and guide the focus of the researchers’ work. The NIHR suggest people with a lived experience of a condition can help shape and refine research questions not already commissioned by funders.^5^ NIHR also state that: ‘It is important that you describe in as much detail as possible how patients and the public have been involved in the development of the application as well as plans for involvement in the potential research’.^6^ This imperative to instigate PPI at the earliest stages of research was further cemented by a recent Call to Action comprising three HTA expert commentaries, in which it was suggested that all HTA should involve patients from beginning to completion.^7^

The benefits of including PPI within research are well documented. For public contributors, involvement in research can instil a sense of empowerment, of being valued, and help develop new skills, while researchers can gain a greater understanding of lived experiences within their research area.^8^ In a qualitative study in the United Kingdom, it was noted that PPI has a positive impact on developing research questions and ideas, as well as a selection of outcome measures for assessment.^9^ Currently, guidance on how to involve people in research more generally is available from organisations such as the NIHR, while a tool to transparently report the involvement and impact of patients in research is available in the form of the GRIPP2 guideline.^10^ There is also guidance for researchers on how to include PPI within specific types of research. For example, the Authors and Consumers Together Impacting on eVidencE (ACTIVE) framework aids researchers undertaking systematic reviews to think about the way in which PPI is embedded into the process.^11^ There are also different methodologies outlined to identify priority research questions such as the James Lind Alliance (JLA) approach.^12^, ^13^ Here, we do not describe the identification of priority questions for pelvic organ prolapse (POP) research; rather, we discuss the refinement of research objectives, outcomes, and methods through public involvement. Given this, we draw on, but do not replicate, interpretive assessment approaches where consensus is reached with purposeful domination by public voice. However, defined practical guidance on when and how best to involve people when preparing proposals for HTA is currently not easily accessible and not tailored to the grant application process. This poses a problem for researchers involved with HTA wishing to involve people with a lived experience in the formulation of their research questions, rationale and outcome measures.

For our potential HTA, we considered assessing the clinical effectiveness and cost‐effectiveness of nonsurgical interventions for treating POP in women, using an evidence synthesis approach with a health economic analysis to estimate the cost‐effectiveness of the treatments. POP refers to the descent of a woman's pelvic organs (the uterus, bladder or rectum) into the vagina.^14^ A survey of UK General Practice found that 8.4% of women reported having a vaginal bulge or lump,^15^ suggesting that the condition is highly prevalent within the United Kingdom. Although the National Institute for Health and Care Excellence (NICE) consider nonsurgical interventions as first‐line treatment for POP,^16^ surgery is also an option. However, in July 2018 the UK Government announced a ‘high vigilance restriction’ on the use of surgical mesh to treat POP and urinary incontinence and, in June 2019, NICE withdrew recommendations to use synthetic polypropylene or biological mesh for women with recurrent anterior vaginal wall prolapse.^16^ Following this, The Cumberledge Report noted that women want to be better informed about their treatment choices for POP.^17^

We therefore believed it was imperative to involve women with a lived experience of POP early in the process of writing our proposal for an HTA, so their views could shape the objectives and methods of the work. Here, we describe our experience of involving people with a lived experience of POP in developing and defining our proposal, specifically the open recruitment of two co‐applicants with POP to help govern the project and the design and facilitation of a workshop including women with a lived experience of POP. We also describe how we incorporated the UK Standards for Involving Patients into our proposal development, give reflections on the process undertaken and highlight potential recommendations for future work in the area.

## 2 AIM

To report the process for incorporating PPI into an HTA proposal surrounding POP.

## 3 METHODS

### 3.1 Theoretical considerations

The PPI activities were underpinned by principles defined within the ACTIVE framework.^11^ Although the ACTIVE framework is primarily designed to help describe PPI in different stages of a full systematic review, we used the framework to help conceptualise how PPI could be involved in planning the HTA, particularly as its first two listed stages (developing a question and planning methods) are directly relevant to development of an HTA proposal. The principles of the ACTIVE framework applied in our work are detailed in Table 1. Additionally, throughout the design, implementation and evaluation of our PPI workshop, we were mindful of the UK Standards for Involving Patients.^3^, ^4^ A summary of how we adhered to the individual standards is given in Table 2. The GRIPP2 reporting checklist for this paper is also available in Supplementary File 1.^10^

**Table 1:** Principles of ACTIVE framework undertaken.

Framework construct	Category
Who was involved?	Patients, carers and/or their families
How were the stakeholders recruited?	Open, fixed (for patient co‐applicants) Open, flexible (for workshop)
What was the mode of involvement?	Approach: continuous Method: direct interaction
At what stage in the review process did involvement occur?	1. Develop question 2. Plan methods
What was the level of involvement (at each stage)?	1. Develop question: influencing 2. Plan methods: controlling

Abbreviation: ACTIVE, Authors and Consumers Together Impacting on eVidencE.

*Source*: Adapted from Pollock et al.^11^

**Table 2:** UK Standards for Public Involvement in relation to the development of the HTA proposal.

UK Standard	Summary of standard	Activities
Inclusive Opportunities	Offering public involvement opportunities that are accessible and reach people and groups according to research needs	‐ Involving PPI within the development of the proposal ‐ Widely advertising the co‐applicant opportunity through different organisations and social media ‐ Advertising of opportunity to attend the workshop on the VOICE platform ‐ Offering honoraria to participants for their time
Working Together	Work together in a way that values all contributions, and that builds and sustains mutually respectful and productive relationships	‐ Ensuring the purpose of the workshop was clear ‐ Recognition of the role of the patient co‐applicants and workshop attendees in shaping the proposal
Support and Learning	Offer and promote support and learning opportunities that build confidence and skills for public involvement in research	‐ Giving support to PPI members through PPI Lead and Co‐PI ‐ Inducting co‐applicants to project ‐ Evaluation of the workshop and taking feedback forward to other activities
Communications	Use plain language for well‐timed and relevant communications as part of involvement plans and activities	‐ Flexibility in communication methods (email, Zoom) ‐ Gathering feedback in person and via evaluation forms ‐ Inclusion of patient co‐applicants in research team correspondence
Impact	Seek improvement by identifying and sharing the difference that public involvement makes to research	‐ Clearly signposting contribution of PPI to the research plan within the potential HTA ‐ Reflection and evaluation of PPI involvement ‐ Communicating impact of PPI to workshop attendees
Governance	Involve the public in research management, regulation, leadership and decision making	‐ Patient co‐applicants help to govern the HTA proposal process ‐ Inclusion of patient co‐applicants in research team meetings ‐ Respect for voices and experiences of PPI ‐ Defined plan in place for PPI involvement within the proposal process and beyond ‐ Resources in place to give honoraria to PPI contributions and PPI activities built into capacity for the proposal ‐ Ensuring confidentiality when embedding PPI experiences into the proposal

Abbreviations: HTA, Health Technology Assessment; PI, principal investigator; PPI, patient and public involvement.

*Source*: Adapted from Crowe et al.^4^

### 3.2 Resourcing of PPI activities

Paying the public fairly for their time working on research is part of the ‘Inclusive Practice’ UK standard for public involvement.^3^ However, many researchers do not have access to funding whilst developing an HTA proposal. While having access to funding has been noted to be a key facilitator for PPI activities when preparing proposals,^18^ it has also been noted that obtaining funding is often difficult.^19^

To fund our PPI activities, we approached the North East and North Cumbria Research Design Service (RDS NENC) to apply for a Patient Involvement Fund (PIF) award. We detailed our plans for PPI within and beyond the potentially funded project, answering queries upon our application from members of the RDS NENC Consumer Panel and RDS NENC team. Subsequently, £500 of funding was made available to grant honoraria for PPI contributions at the rates recommended by the NIHR.^20^ Some of the funding went to resourcing activities undertaken by our PPI co‐applicants, including attending meetings and reviewing documentation. Funding was also set aside to reimburse the participants in our PPI workshop for their time.

### 3.3 Recruitment and selection of PPI co‐applicants

After securing funding, we proceeded to open recruitment of a PPI co‐applicant. The role of the co‐applicant on the proposal was intended to ensure governance of the project from the patient perspective; they would be responsible for ensuring that the patient voice was heard throughout the proposal and subsequent funded project, as per the UK Standard.^3^, ^4^

We developed an advert spanning a single side of A4 explaining the background to the project, the expectations of the role as defined by NIHR guidance (e.g. helping to develop the study and overseeing its progress and conduct),^21^ what the co‐applicant could expect to be involved in and potential training and support opportunities offered by the research team. We also set out the essential criteria for the role: applicants must have had a lived experience of POP (either as a patient or as a family member or carer of someone with POP) and some prior experience of being involved in research. However, they did not have to have specific experience in evidence syntheses, cost‐effectiveness analyses or HTA. We felt that recruiting someone who already had some prior experience with research would enable them to feel more comfortable taking on the aspects of governance expected of the role of a patient co‐applicant.

The advert was checked for comprehensibility by members of the RDS consumer panel and amended appropriately before being distributed via VOICE, the Royal College of Obstetrics and Gynaecologists’ Women's Network, and via Newcastle University's Health Economics and Evidence Synthesis Group Twitter feed. We also attempted to contact local women's health groups regarding the opportunity (e.g. via Facebook) to help ensure the recruitment process was as open as possible.

We received 14 applications for the initial position, which were anonymised and reviewed by two researchers. The decision of whom to speak with was based on the strength of their prior research experience, as we felt they would best understand the governance and oversight role of the co‐applicant and be able to help bring the patient perspective to light with support and guidance from the project's PPI Lead.

Following the workshop (see further details below), the two researchers, including the PPI Lead, discussed recruiting a second PPI co‐applicant to help further ensure the voice of those with POP was embedded into the core research team and that the burden of responsibility for governing PPI during the proposal and the subsequent funded project would not fall on a single person. A second potential co‐applicant, who had attended the workshop, was directly approached via email about the opportunity.

### 3.4 Involvement of PPI co‐applicants in the research proposal

An initial, informal induction was held with each co‐applicant to further discuss the aims of the potential projects, the timelines for delivery, expectations of the role, and opportunities for further questions. They were each also given the opportunity to highlight any areas where they would potentially like more hands‐on research experience, involvement, or additional support, so this could be formally costed into the HTA proposal as needed.

Both co‐applicants were given the opportunity to comment on the full research proposal, once drafted, and for their comments to be incorporated alongside those from other team members. They were also invited to attend a research team meeting to discuss the proposal. During this meeting, they were actively encouraged by the facilitators to ask for clarification of points that were not clear or contained technical jargon, as well as to give their perspectives on topics being discussed.

### 3.5 PPI workshop recruitment

Due to pragmatic time constraints, advertising of the workshop was more limited compared to the recruitment of the patient co‐applicants; we only recruited participants by advertising the session via the VOICE platform. We used plain language to describe: the purpose of the workshop; that the attendees need to have a lived experience of POP but that research experience was not essential; the time commitment expected; and the reimbursement attendees would receive. We received 10 expressions of interest but two of these expressions of interest were considered ineligible as they were from people who did not have a lived experience of POP. We wrote separately to these two people to inform them of this decision. We invited the remaining eight women to participate in the workshop but only four attended.

### 3.6 PPI workshop conduct

A two hour workshop for women with a lived experience of POP and their carers was run in October 2021. The intention of the workshop was to elicit the views of people with a lived experience on the following: what research into POP should consider; any concerns or hopes that they had about the HTA project we were proposing; what they considered to be the most meaningful clinical outcomes for assessment; and their experience of the care pathway. These topics were chosen to ensure that views were heard on the three key components of the HTA: the rationale and overall scope of the project; the evidence synthesis methods; and the foundation of the economic evaluation.

**Figure 1:**
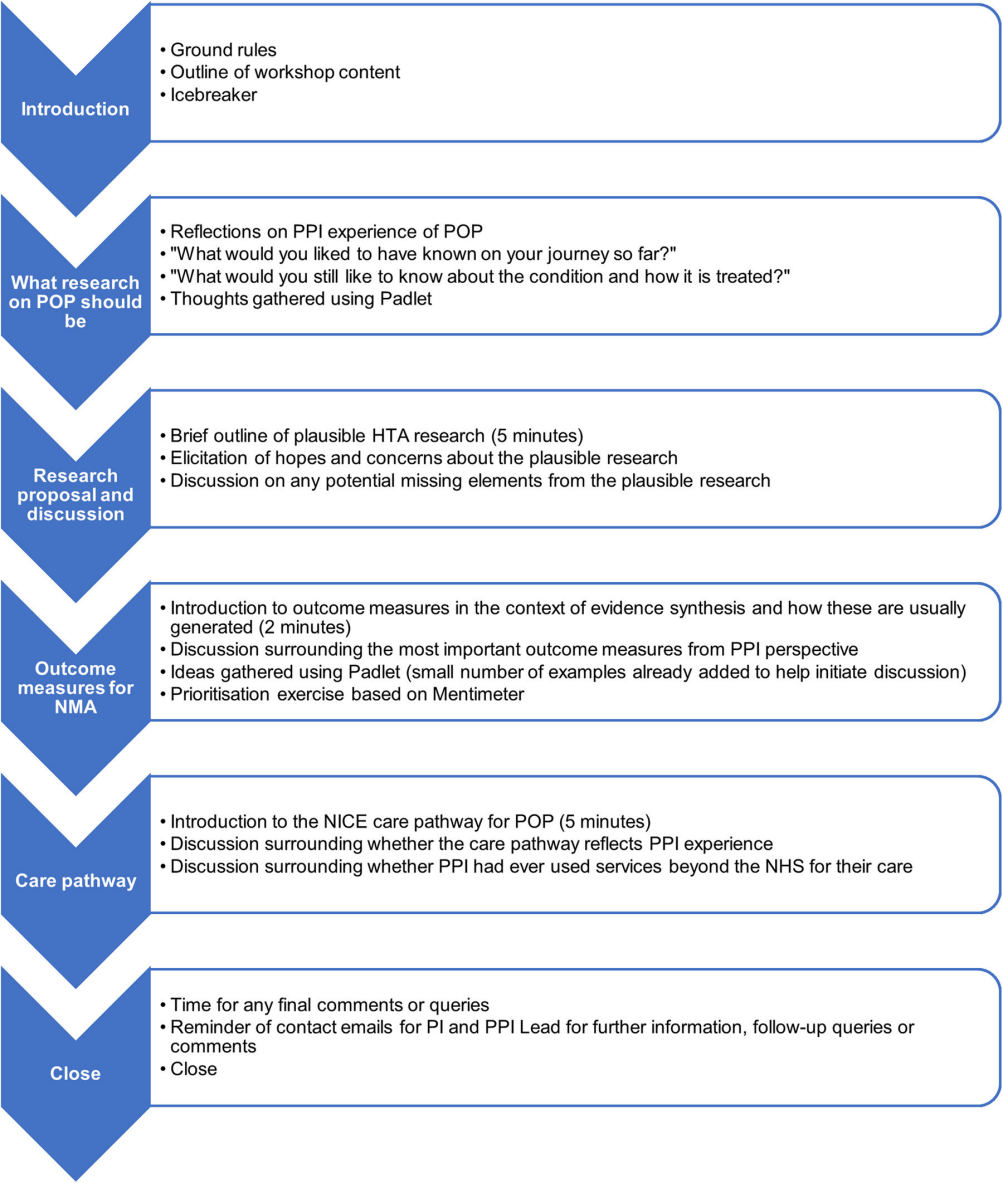
Structure and content of PPI workshop. HTA, Health Technology Assessment; NICE, National Institute for Health and Care Excellence; NMA, network meta‐analysis; PI, principal investigator; POP, pelvic organ prolapse; PPI, patient and public involvement.

Two researchers facilitated the workshop. The aim of this approach was to ensure that one researcher's voice did not dominate the conversation. We used Padlet, an online platform that allows sharing of brief posts on a virtual ‘wall’, to document the views of participants in real time. We also planned to utilise Mentimeter, an online polling system, to prioritise outcome measures for the evidence synthesis. The outcome measures to rank consisted of examples given from already‐published work on POP and were added to in real‐time based on responses from workshop attendees. We hosted the workshop via Zoom, to allow participants from a wide geographical area to attend. Given this, we kept the use of PowerPoint slides to a minimum and turned screen sharing off whenever possible to instil a greater sense of community between researchers and participants, as well as encourage active participation.^22^ The planned structure and content of the workshop are detailed in Figure 1.

As demonstrated in Figure 1, during the development of our workshop, we attempted to embed as many spaces for people to share their opinions and experiences as possible, with as little speaking from the research team as possible. This reflects what Knowles et al describe as authentic involvement resulting from both the ‘space to talk’ and the ‘space to change’ (p. 5).^23^ In their view, ‘space to talk’ is allowing all members to have time to talk and recognise lived experience as being expert opinion, while ‘space to change’ is acting on shared knowledge, with the flexibility to alter study methods and scope (p. 5).^23^ The ways in which we promoted the ‘space to talk’ and the ‘space to change’, adapted from Knowles et al., are demonstrated in Table 3.^23^

**Table 3:** Embedding of spaces to talk and change within and beyond the workshop.

	Space to talk	Space to change
Design	‐ Designing the format as a workshop and not a meeting ‐ Explicitly prioritising lived experience so that it was recognised as being a form of expert knowledge	‐ Reactive to thoughts and concepts ‐ Using prompts and examples to help elicit ideas ‐ Collecting ideas in ‘real time’ using Padlet ‐ Introducing the use of Mentimeter to prioritise outcome measures, but dropping this element based on feedback ‐ Removing the section regarding the care pathway from the workshop to allow more space to discuss openly about the other topics ‐ Discussion of potential dissemination outlets
Researcher‐attendee relationship	‐ Inviting PPI as equal experts on the topic ‐ Ensuring enough space for PPI attendees to discuss their views and discuss issues with each other	‐ Willingness of researchers to adapt methods and rationale behind the HTA proposal to meet the needs and priorities of PPI
Institutional process	‐ Providing reimbursement for attendance at the workshop ‐ Providing feedback on the proposed activities	‐ Responsive to any requests to change the mode of reimbursement

Abbreviations: HTA, Health Technology Assessment; PPI, patient and public involvement.

*Source*: Adapted from Knowles et al.^23^

By being mindful of ‘space to change’ during the workshop, we changed the format of the workshop dynamically. First, the discussion surrounding POP research, our potential research plan and the outcome measures took longer than originally anticipated, as the attendees had much to offer and we wanted to give space for these aspects to be explored at length. As such, we did not discuss the care pathway as originally intended. We also elicited opinions on where the attendees felt the research could be shared and where they usually go to find out about health‐related topics. Although not originally planned, this impacted our dissemination plans for the potential HTA.

### 3.7 Impact of PPI workshop on proposal

**Figure 2:**
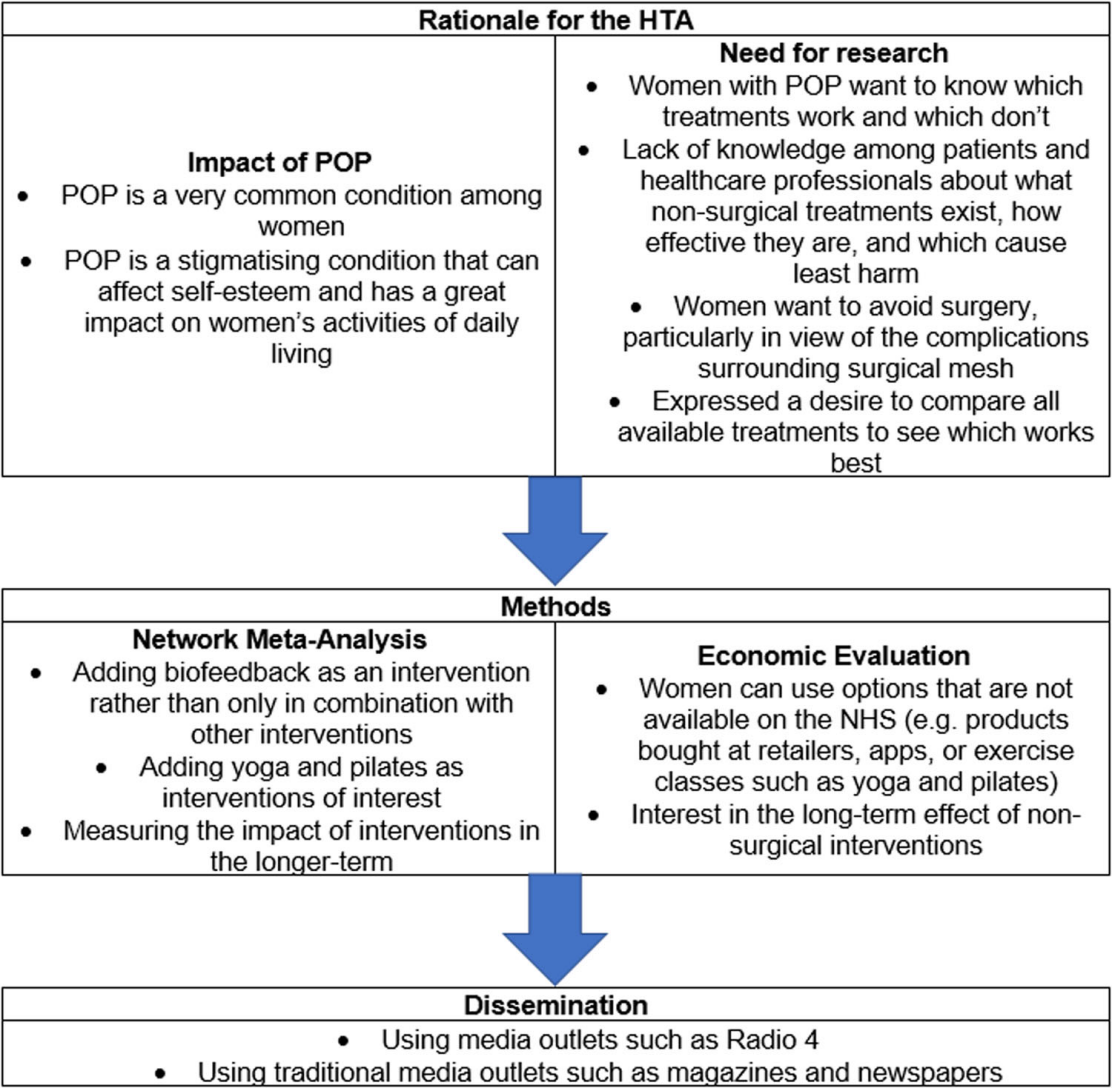
Impact of PPI workshop on potential HTA. HTA, Health Technology Assessment; NHS, National Health Service; POP, pelvic organ prolapse; PPI, patient and public involvement.

The ways in which the PPI workshop influenced and impacted our potential research are detailed in Figure 2. The elements described in Figure 2 were all either added to our methods or used to help further define the rationale for undertaking an HTA on the topic of nonsurgical interventions for treating POP.

### 3.8 PPI workshop evaluation

During the PPI workshop, we asked for any comments and feedback regarding the timing of the workshop. One participant noted it would be more convenient to host any future events in the evenings or on weekends; we will take this feedback on to other involvement opportunities including those with POP.

After the workshop, we sought feedback from the attendees via a short online feedback form; three of four attendees responded. In an open‐ended question, respondents expressed that they appreciated the space to discuss the issue of POP and their opinions, which also acted as a helpful support mechanism for the group and made them feel less socially isolated. Additionally, one respondent noted that they thought the team listened to the views of the attendees and felt their experiences were valued and would be of use within the research. Furthermore, a different respondent noted that our decision to relay back information we had received helped them feel like they were valued and listened to. The feedback demonstrates the positive impact implementing ‘space to talk’ and ‘space to change’ had on the PPI attendees, as well as our HTA proposal.^23^

Building on this, it has been noted that some PPI participants wish to receive individual feedback regarding their impact, as this may improve contributions and their motivation to stay involved.^9^ As such, following the workshop and evaluation process we sent all participants an information sheet informing them of the ways in which we used their views and experiences to inform our potential HTA. Although this was not individualised feedback, we felt this helped to communicate the impact the workshop had on the development of the proposal and would help motivate participants to continue being involved in the project going forward.

## 4 DISCUSSION

### 4.1 Reflections on impact of PPI workshop on the proposal

We believe that PPI greatly enhanced the scope, rationale and methods of our potential HTA. Although we were not able to cover all the initially planned components, by listening to the experiences of women with a lived experience of POP and allowing them to have the space to comment on and interrogate our ideas, we believe the development of our potential HTA was strengthened and focused more on the needs of service users. In particular, the experiences of those with a lived experience of POP strengthened the rationale for pursuing further research into nonsurgical interventions for POP.

### 4.2 Difficulties of PPI whilst developing research proposals

It must be noted that the process of involving PPI at this early stage in the grant‐writing process was very resource‐intensive. The labour involved in supporting the involvement of patients and the public in research and the lack of practical support to undertake this work has been previously described,^8^, ^24^ and was also the case with this proposal. Neither of the researchers who jointly developed and facilitated PPI activities for this application were funded to undertake this work. Both worked on the PPI activities within the proposal while also maintaining work on other, funded projects. This had an impact on the level of involvement we were able to embed when developing our proposal. For example, we decided not to discuss the care pathway underpinning the potential economic evaluations in detail within the workshop and we did not have the capacity and resources to discuss this further, either in another workshop or another activity. We planned to revisit this aspect of the proposed research with a PPI Advisory Group should our proposal have been funded. However, it meant the impact of PPI on our planned economic evaluation was limited within the proposal.

The lack of support may make embedding PPI to the level we have within this proposal challenging for other potential HTAs. The level of PPI activity considered appropriate for potential HTAs may need to be assessed on a case‐by‐case basis but, so far, there is no guidance or decision aid on when and how to implement PPI.

Although we attempted to ensure that the initial recruitment of a patient co‐applicant was as open as possible, this was not the case with the recruitment of a second co‐applicant, who was approached directly due to pragmatic time constraints. This may have potentially caused a bias in the selection process, as we did not give others (including those from under‐served communities) the opportunity to apply for this second co‐applicant role.

We also did not undertake a fully open process for recruiting for the PPI workshop, as we only advertised the opportunity via a single platform (VOICE). We may have elicited responses from a wider range of lived experiences had we advertised more widely. Although we invited eight women to the workshop initially, only four were able to attend. This was, however, considered a strength of the workshop by its participants, as they noted that they had more time to discuss and express their views openly with each other. Additionally, we held the workshop online and on a weekday morning. This could mean that we excluded the potential participation of people who had other responsibilities (e.g. work or care commitments), as well as precluding the participation of anyone without access to the Internet or with less confidence in using online meeting platforms.

Crucially, we began engaging and involving patients and members of the public, including our patient co‐applicants, after initial discussions on the scope and methods of the project had already begun amongst researchers. We reflected on people's experience of POP, what they would you liked to have known on their journey so far, and what they would still like to know about the condition or how it is treated. We then consulted on our potential HTA scope with the PPI participants in our workshop and asked for feedback, concerns or issues, rather than asking further open questions regarding whether we were asking the right question or the one of most importance to them. This could constitute what Green et al describe as a ‘co‐opted’ relationship between patients, the public and researchers, whereby patient members are added into an already‐existing framework (pp. 5–6).^25^ To ameliorate this, we gave participants space to present their views on what research into POP should be about before presenting our potential HTA and eliciting views on this. We also tried to express that it was valid for attendees to disagree with the scope or method of research we were proposing.

### 4.3 Reflections on embedding PPI in future research proposals

Although PPI input is undoubtedly valuable to grant proposals, researchers need to weigh the balance between their time investment in PPI activities when developing an HTA and the potential gains of investing this time. Research into engaging PPI should focus on involvement at this early stage in the process so that researchers can better decide what type and level of involvement for their potential HTA are meaningful to patients and the wider public, as well as how to implement this. This could initially take the form of a decision aid to help researchers identify when a greater level of PPI input may be required. This decision aid should be designed and implemented in collaboration with PPI representatives to ensure that its content and use are responsive to their needs and is less researcher‐driven. An open‐access repository of tools to help researchers embed PPI into the process may also be useful. Practical guidance on how best to undertake PPI activities to shape HTA proposals would also be welcomed, so that researchers have the tools to undertake PPI while still being mindful of time constraints. Researchers may also wish to reflect on the NIHR INCLUDE project guidance as a framework for involving people from marginalised and under‐served groups in research.^26^

## 5 CONCLUSIONS

Our workshop demonstrated that involving patients and/or the public, and being receptive to their experiences and opinions, can have a very positive impact on the scope and methods of a stage one HTA proposal. However, researchers need to be mindful of the resource required to properly plan and facilitate PPI activities. Work to develop decision aids to help researchers decide how best to involve PPI at the earliest stages of an HTA should be conducted.

## AUTHOR CONTRIBUTIONS

Eugenie Evelynne Johnson assisted with recruiting participants, design and facilitation of the workshop; wrote the manuscript. Joanne Lally assisted with the design of the workshop; assisted with the writing of the manuscript. Allison Farnworth assisted with the design of the workshop; assisted with the writing of the manuscript. Fiona Pearson assisted with recruiting participants, design and facilitation of the workshop; assisted with writing the manuscript. All authors read and approved the final manuscript.

## CONFLICT OF INTEREST STATEMENT

The authors declare no conflict of interest.

## Supporting information

Supplementary information.

## Data Availability

The data that support the findings of this study are available from the corresponding author upon reasonable request.
